# Need for numbers: assessing cancer survivors’ needs for personalized and generic statistical information

**DOI:** 10.1186/s12911-022-02005-2

**Published:** 2022-10-05

**Authors:** Ruben D. Vromans, Saar Hommes, Felix J. Clouth, Deborah N. N. Lo-Fo-Wong, Xander A. A. M. Verbeek, Lonneke van de Poll-Franse, Steffen Pauws, Emiel Krahmer

**Affiliations:** 1 Department of Communication and Cognition, Tilburg Center for Cognition and Communication, Tilburg School of Humanities and Digital Sciences, Tilburg University, P.O. Box 90153, 5037 LE Tilburg, The Netherlands; 2 Department of Research and Development, Netherlands Comprehensive Cancer Organisation, Utrecht, The Netherlands; 3 Department of Statistics and Methodology, Tilburg School of Behavioral Sciences, Tilburg University, Tilburg, The Netherlands; 4 Department of Medical Psychology and Psychotherapy, Erasmus University Medical Center, Rotterdam, The Netherlands; 5 Department of Medical and Clinical Psychology, Tilburg School of Behavioral Sciences, Tilburg University, Tilburg, The Netherlands; 6 Division of Psychosocial Research and Epidemiology, The Netherlands Cancer Institute, Amsterdam, The Netherlands; 7 Collaborative Care Solutions, Philips Research, Eindhoven, The Netherlands

**Keywords:** Cancer statistics, Patient-centered healthcare, Patient information needs, Personalization, Risk communication, Shared decision-making, Tailoring

## Abstract

**Background:**

Statistical information (e.g., on long-term survival or side effects) may be valuable for healthcare providers to share with their patients to facilitate shared decision making on treatment options. In this pre-registered study, we assessed cancer survivors’ need for generic (population-based) versus personalized (tailored towards patient/tumor characteristics) statistical information after their diagnosis. We examined how information coping style, subjective numeracy, and anxiety levels of survivors relate to these needs and identified statistical need profiles. Additionally, we qualitatively explored survivors’ considerations for (not) wanting statistical information.

**Methods:**

Cancer survivors’ need for statistics regarding incidence, survival, recurrence, side effects and quality of life were assessed with an online questionnaire. For each of these topics, survivors were asked to think back to their first cancer diagnosis and to indicate their need for generic and personalized statistics on a 4-point scale (‘not at all’- ‘very much’). Associations between information coping style, subjective numeracy, and anxiety with need for generic and personalized statistics were examined with Pearson’s correlations. Statistical need profiles were identified using latent class analysis. Considerations for (not) wanting statistics were analyzed qualitatively.

**Results:**

Overall, cancer survivors (*n* = 174) had a higher need for personalized than for generic statistics (*p* < .001, *d* = 0.74). Need for personalized statistics was associated with higher subjective numeracy (*r* = .29) and an information-seeking coping style (*r* = .41). Three statistical need profiles were identified (1) a strong need for both generic and personalized statistics (34%), (2) a stronger need for personalized than for generic statistics (55%), and (3) a little need for both generic and personalized statistics (11%). Considerations for wanting personalized cancer statistics ranged from feelings of being in control to making better informed decisions about treatment. Considerations for not wanting statistics related to negative experience with statistics and to the unpredictability of future events for individual patients.

**Conclusions:**

In light of the increased possibilities for using personalized statistics in clinical practice and decision aids, it appears that most cancer survivors want personalized statistical information during treatment decision-making. Subjective numeracy and information coping style seem important factors influencing this need. We encourage further development and implementation of data-driven personalized decision support technologies in oncological care to support patients in treatment decision making.

**Supplementary Information:**

The online version contains supplementary material available at 10.1186/s12911-022-02005-2.

## Background

When patients diagnosed with cancer are making a decision about treatment, they need to be informed about the associated risks and benefits of treatments. To support this, healthcare providers could share statistical information related to outcomes of treatments (e.g., survival benefits, cancer free survivorship) and the risks of adverse effects (e.g., side effects, impact on quality of life) in order to facilitate shared decision-making [1, 2]. However, it might be hard for patients to apply statistics to their individual situation, since those are often *generic* and based on *all* patients diagnosed with a certain type of cancer [3, 4]. So, when a 45-year-old man, for example, is diagnosed with prostate cancer, generic statistics may be of limited value since they are derived from the entire group of prostate cancer patients, consisting of mostly substantially older men, whose data was obtained from randomized controlled trials or observational datasets. With the increased availability of medical and patient reported outcome data, more *personalized* statistics can be provided by comparing individual patient and disease characteristics (e.g. tumor type, stage, age, gender) with specific patient groups with similar characteristics, thereby providing patients with more specific and personalized probability information of a certain outcome [5, 6]. In the case of the 45-year old male with prostate cancer, his data could be compared with a subset of comparable men, typically younger ones, which in turn may lead to more accurate risk perceptions and informed decision-making [7]. However, there is also a potential downside to this: since the statistics are more personally relevant for the 45-year old male, they might conceivably also induce more anxiety in him, especially when the numbers are not positive, and perhaps, for this reason, the more generic statistics would be preferred. In truth, we know very little about who would want personalized statistics under which circumstances, and the increasing availability of this kind of information raises a number of new as yet unanswered questions. To what extent do patients want to receive personalized and/or generic statistics? And are these different needs related with any personal or psychosocial characteristics?

However, assessing patients’ statistical information needs is challenging, especially since communicating statistics (and especially personalized ones) in clinical practice remains limited [8, 9]. Healthcare professionals often do not communicate such numbers due to time constraints [10], data unavailability [11, 12], unreliable data (selection bias in observational data), or fear of disrupting patients’ hope [13]. Additionally, clinical decision-support systems that use personalized data to inform decisions are often not rigorously tested, which means that the impact on patient care remains unknown [14]. Even if clinical support systems are evaluated, this happens in their specific clinical context, making it difficult to draw general conclusions about the usage of personalized data in healthcare [15]. In the same vein, most decision aids for patients with cancer facing treatment decisions do not contain personalized statistics either, or do not contain any numerical information at all [16–18]. This makes it difficult to assess whether and in what circumstances patients are open to receiving personalized statistics during treatment decision-making.

Even though several survey studies repeatedly suggest that patients have a desire for receiving prognostic information in general [19–21], there has been no detailed investigation into patients’ need for specifically receiving personalized numbers and statistics for a range of different outcomes. A recent qualitative study found suggestive evidence that majority of cancer patients want to receive personalized cancer statistics such as survival rates or treatment side effects risks [22], but a more systematic and quantitative analysis is lacking. Therefore, the first aim of this study is to quantitatively assess the extent to which patients have a need for personalized or generic statistics after a cancer diagnosis. Based on previous research regarding patients’ (prognostic) information needs, we hypothesize that there is a need for both generic (H1a) and personalized (H1b) statistics.

If we assume that personalized statistics are available to both healthcare providers and patients, there are several challenges to overcome, both in consultations and (online) patient decision aids. First, patients differ in how much information they want to receive, also known as *information coping style* [23]. Some patients desire detailed and more voluminous information (information-seekers), whereas others prefer to receive little or minimal information (information-avoiders). Therefore, we may expect that information-seekers would want both generic and personalized statistics, whereas information-avoiders prefer to avoid both. Second, interpreting risks and probabilities seems to be problematic for many [24]. At the same time, we cannot avoid numbers as risk communication research strongly recommends to communicate risks in numbers (e.g. “1 out of 10 people experience side effect X”) instead of words-only (e.g. “it is unlikely”) [4, 25–27]. That is why *subjective numeracy* should be considered when investigating the need for personalized and generic statistics, with the expectation that people with higher subjective numeracy have a higher need for personalized statistics than those with lower subjective numeracy [28]. Third and finally, as patients diagnosed with cancer often experience *anxiety*, which can in turn influence their general need for information, we expect that anxiety will also be negatively related to cancer patients’ need for especially personalized statistics [19, 20, 29]. Since evidence on the relationship with generic and personalized needs and all these factors (information coping style, subjective numeracy, and anxiety) is scarce, no formal hypotheses were formulated. These all relate to the second aim of our study: to explore different patient factors that could influence their need for generic and personalized statistics.

The third aim of this study is to identify statistical need profiles. Similar to earlier research, we seek to explore the more complex patterns underlying patients’ needs for generic and personalized statistics into statistical needs profiles [30]. We expect that there might be several factors (cancer type, age, information topic, anxiety, information coping style, numeracy, gender) that could all have an impact on to what degree patients want to receive generic and/or personalized statistics [22, 30–32].

Our fourth and final aim is to explore reasons people have for (not) wanting to receive personalized or generic statistical information after a cancer diagnosis. It is currently unknown what reasons patients have for not only receiving personalized statistics, but also why they still want generic statistics. Knowing more about the underlying factors (aim 2) and views (aim 3) could help doctors identify those patients that might want personalized or generic statistics, and those that do not.

All hypotheses and expectations were pre-registered within the Open Science Framework prior to data collection (https://osf.io/qv35z/).

## Methods

### Sample and procedure

In April 2020, 664 cancer survivors with breast, colon, lung, or prostate cancer were invited to participate. Cancer survivors were recruited from a Dutch panel (Kanker.nl). Participants eligible for participation received an invitation to enter the study via e-mail. Participation was voluntary and no reminders were sent out to avoid overburdening the panel. Sociodemographic, disease-related questions, and statistical information needs (SIN) were assessed in a newly developed questionnaire, also examining information coping style, subjective numeracy and anxiety level, and lasted about 20 min. The complete questionnaire (Dutch and English) is publicly available https://osf.io/qv35z/.

### Measures

#### Socio-demographic and clinical factors

Demographic and clinical variables included age, gender, education level, marital status, having children, employment status, tumor type, year of diagnosis, and primary treatment(s).

#### Need for personalized and generic statistics

The need for personalized and generic statistics was assessed by a newly developed SIN-instrument. First, an explanation of the difference between a personalized and a generic statistic was provided, followed by a control question to check whether participants understood the difference (*n*_wronganswer_ = 8/174 (4.6%)). Respondents were then asked to think back to their first cancer diagnosis, and to indicate whether they would have wanted to receive generic and/or specific statistical information regarding: the absolute cancer incidence number (1 item), survival rate (2 items; 5 and 10 year survival rate), treatment-related survival rate (2 items; 5 and 10 year), recurrence rate (2 items; 5 and 10 year), risk of treatment side effects (1 item), and impact of treatment on quality of life (4 items; physical, emotional, cognitive, and social functioning). The selection of topics was based on the needs and preferences of prostate and breast cancer survivors assessed during focus groups [22], and on earlier comparable studies [19, 20, 31]. All items relating to generic statistical needs were combined to create one average generic-SIN score (α = 0.88), and all items relating to personalized statistical needs were used to create an average personalized-SIN score (α = 0.87).

**Fig. 1:**
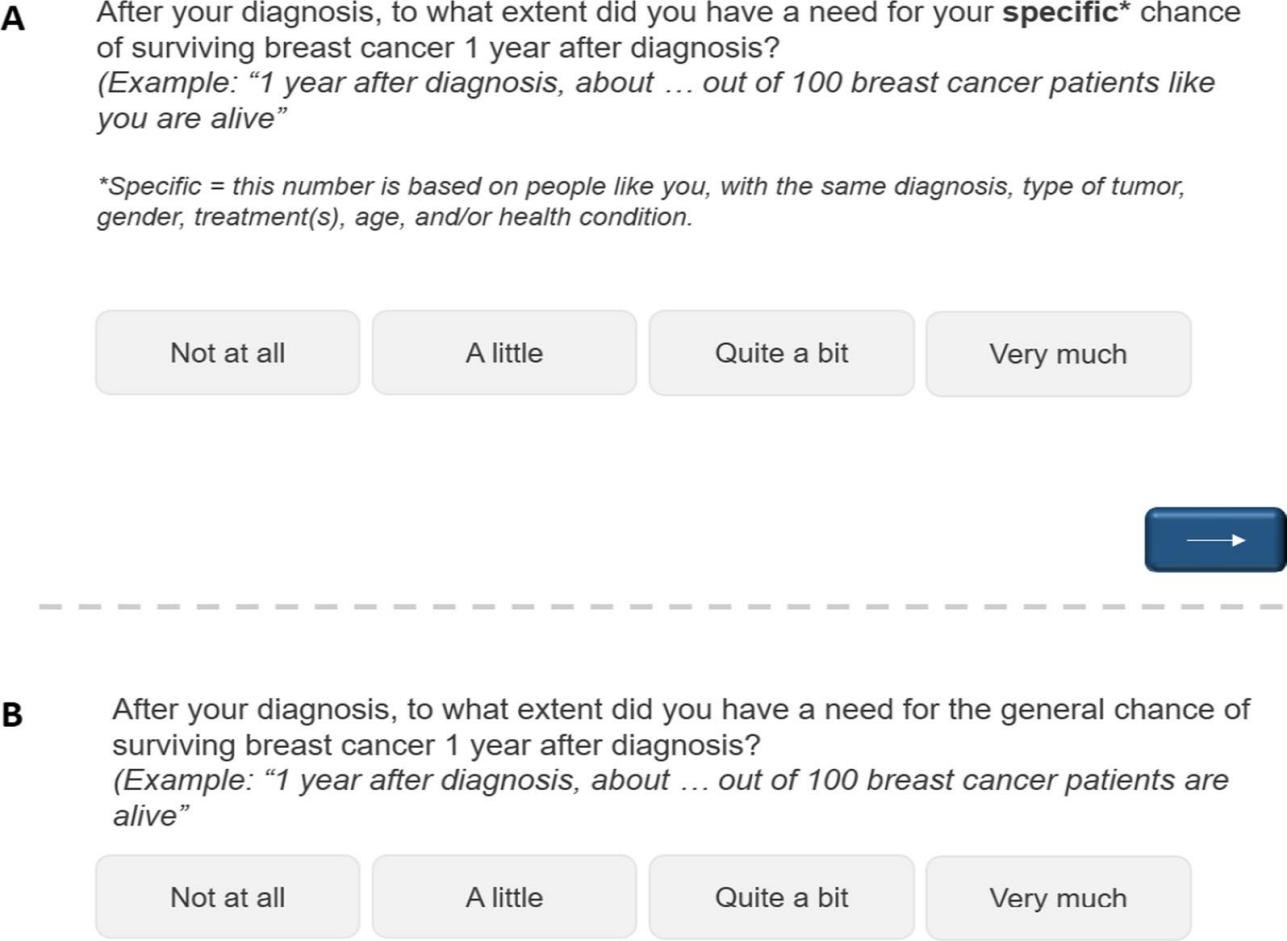
Example items for breast cancer survivors that assess their need for personalized **A** and generic **B** statistics regarding their 1-year survival rate (shown on separate pages)

For each topic, respondents indicated their need for generic and personalized statistics on a 4 point scale (1 = ‘not at all’, 2 = ‘a little’, 3 = ‘quite a bit’, 4 = ‘very much’). These answer categories were taken from the European Organisation for Research and Treatment of Cancer Quality of Life Questionnaire [33]. Each question was clarified with an example, and the questions about the need for personalized statistics included a reminder of what was meant with the term ‘personalized’/’specific’ (Fig. 1). The examples did not include any real data (e.g., the numerator was left out: “… out of 100”), as this might bias participants’ responses. The questionnaire also included an open question where respondents could indicate why they would (not) want to receive personalized/generic statistics The order of personalized and generic statistic items was counterbalanced per topic across all participants. The questionnaire was developed by a team of (health) communication researchers, medical experts in oncology, and a statistician. The instrument was pre-tested^1^ among five patients with cancer regarding understandability, length, clarity and possible missing topics.

#### Information coping style, subjective numeracy, and anxiety level

*Information coping style* was measured with a validated shortened version of the Threatening Medical Situations Inventory [34]. Two styles are distinguished: a monitoring (“information-seekers”) and a blunting information coping style (“information-avoiders”). Assessment was based on two hypothetical descriptions of threatening medical situations, followed by six items assessing to what degree they identify with the statements measured on a 5-point scale (1 = ‘not applicable at all’ and 5 = ‘very applicable’). The internal consistency of the blunting (α = 0.67) and monitoring (α = 0.74) subscales were moderate to good. An information style score was calculated by subtracting the blunting subscale score from the monitoring subscale score, with a higher score indicating a monitoring/information seeker coping style (and a lower score a blunting/information-avoider style) [35–38]. The scales were unrelated to each other (Pearson’s product moment correlation = − 0.08).

*Subjective numeracy* was assessed with the validated, 8-item Subjective Numeracy Scale (SNS) [39, 40], which examines quantitative ability and preference for numerical information measured on a 6-point scale (1 = ‘least numerate’ and 6 = ‘most numerate’) (α = 0.88) [27]. We used the Dutch version of the SNS [41, 42]. The mean subjective numeracy score was determined by computing the average score of the eight items, with higher scores indicating higher subjective numeracy.

*Anxiety level* was assessed with a validated Dutch version of the Anxiety-subscale of the Hospital Anxiety and Depression Scale (HADS) questionnaire [43]. HADS consists of 7 items measured on a 4-point scale (0 = ‘not at all’ and 3 = ‘mostly’) (α = 0.88). Scores were summed, with higher scores representing higher anxiety levels.

### Statistical analyses

We used separate one sample t-tests (test-value: 2^2^) to determine whether cancer survivors had a need for generic statistics and a need for personalized statistics. Comparisons *between* the need for personalized versus generic statistics were tested with separate paired-sample t-tests. For the calculation of effect sizes, Cohen’s *d* was computed, where a *d* of 0.2 represents a small, a *d* of 0.5 a medium, and a *d of* 0.8 a large effect size [44]. We also included confidence intervals. Associations between need for generic and personalized statistics, and information coping style, subjective numeracy, and anxiety level were assessed with Pearson’s correlation coefficients.

An exploratory three-step latent class analysis (LCA) was conducted to identify statistical information needs’ profiles of cancer survivors [45]. All SIN-items (i.e., items on incidence, recurrence, survival, and quality of life) were included as indicators (measurement level was specified as ordinal). The number of classes increased until model fit was sufficient as assessed by the Bayesian Information Criterion (lowest BIC selected), Akaike's information criterion (lowest AIC selected), Consistent AIC (CAIC), and bivariate residuals (lower than 10). The assumption of local independence was relaxed if beneficial for model fit. To compare the classes, differences in information coping style, anxiety level, numeracy, and demographic variables were investigated with Wald tests using the three-step adjustment to account for uncertainty in the classification [45, 46]. Confidence intervals and *p*-values are reported.

The statistical analyses were performed using SPSS statistical software (version 24.0). Tests were 2-sided and considered statistically significant at *p* < 0.05, and adjusted for multiple testing using the Bonferroni correction.

### Exploratory qualitative analysis

We qualitatively analyzed the open-ended question using an inductive thematic analysis [47]. The main purpose of this analysis was to capture broad coding categories for people’s views on (not) wanting generic and/or personalized statistics. We excluded responses that were off topic or that we could not interpret. One researcher (SH) coded each comment, and final themes were discussed between two researchers (SH, RV). Illustrative comments reflecting these themes are included in the results.

### Ethical statement

Ethical approval was granted by the Research Ethics and Data Management Committee (REDC) of the Tilburg School of Humanities and Digital Sciences of Tilburg University (REDC 2020-148a). All methods were carried out in accordance with relevant guidelines and regulations, and the survey protocol was approved by the ethics committee (REDC). All participants gave their digital consent to participate, and the ethics committee approved the use of digital signatures.

## Results

### Sample characteristics

**Fig. 2:**
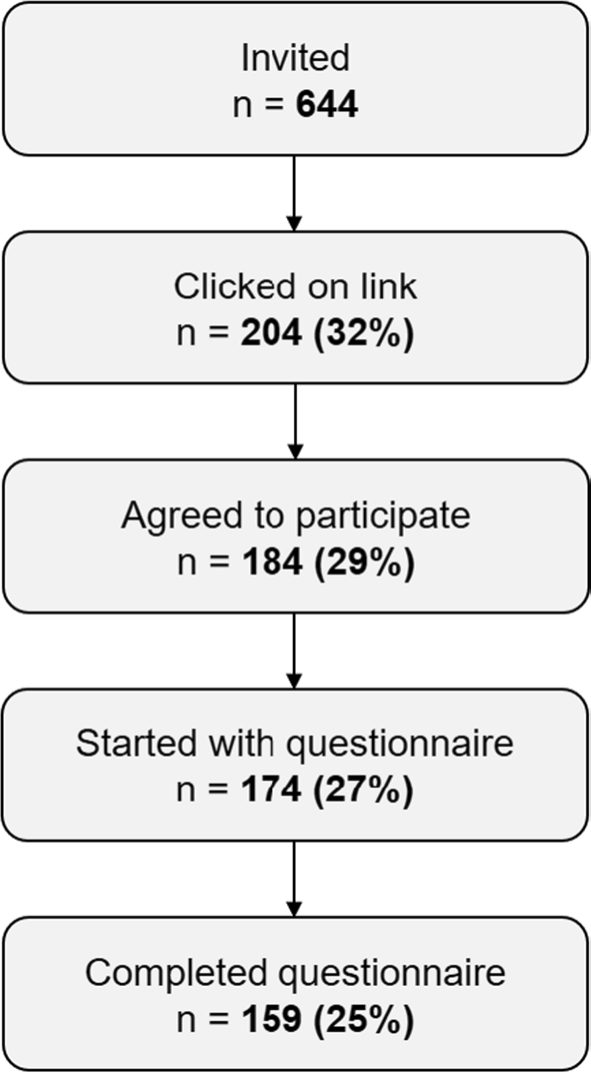
Flowchart of the data collection process

Out of 644 cancer survivors who were invited to participate since they were a member of the Kanker.nl panel, 204 (32%) clicked on the link to launch the survey. Of those, 184 (29%) agreed to participate by giving informed consent. Of those participants, 174 (27%) continued beyond the sociodemographic part of the survey (Fig. 2) and were included in the analyses on SIN. In total, 159 (25%) participants completed the whole questionnaire. Other studies that used the same patient panel had similar response rates [41]. The mean age of the participants was 60.2 years (*SD* = 9.1, median = 60.7) and 59 percent was female (Table 1). The majority of participants (57 percent) had a college/university degree. The mean time since diagnosis was 5.89 years (*SD* = 9.46, median = 3.50).

**Table 1:** Participant characteristics (*n* = 174)

	*n*	%
Gender		
Female	103	59
Male	71	41
Age at time of survey, mean (SD)	60.2 (9.1)	
< 50 years	26	15
50–65 years	90	52
> 65 years	58	33
Education		
Low^a^	15	9
Medium^b^	59	34
High^c^	100	57
Tumor		
Breast	67	39
Colon	40	23
Lung	21	12
Prostate	46	26
Years since first diagnosis, mean (SD)	5.9 (9.5)	
0–5 years	101	58
> 5 years	73	42
Work situation		
Work	56	32
Insurance (ill)	17	10
No work/retired	101	58
Marital status		
Married/living together	138	79
Partner, not living together	2	1
No partner	34	20
Children		
No	50	29
Yes, living with/ living somewhere else	124	71

^a^ Primary and (low levels of) secondary school; ^b^  Secondary school (higher levels) or practical education; ^c^ College and university; SD = standard deviation

### Need for personalized and generic statistics

**Fig. 3:**
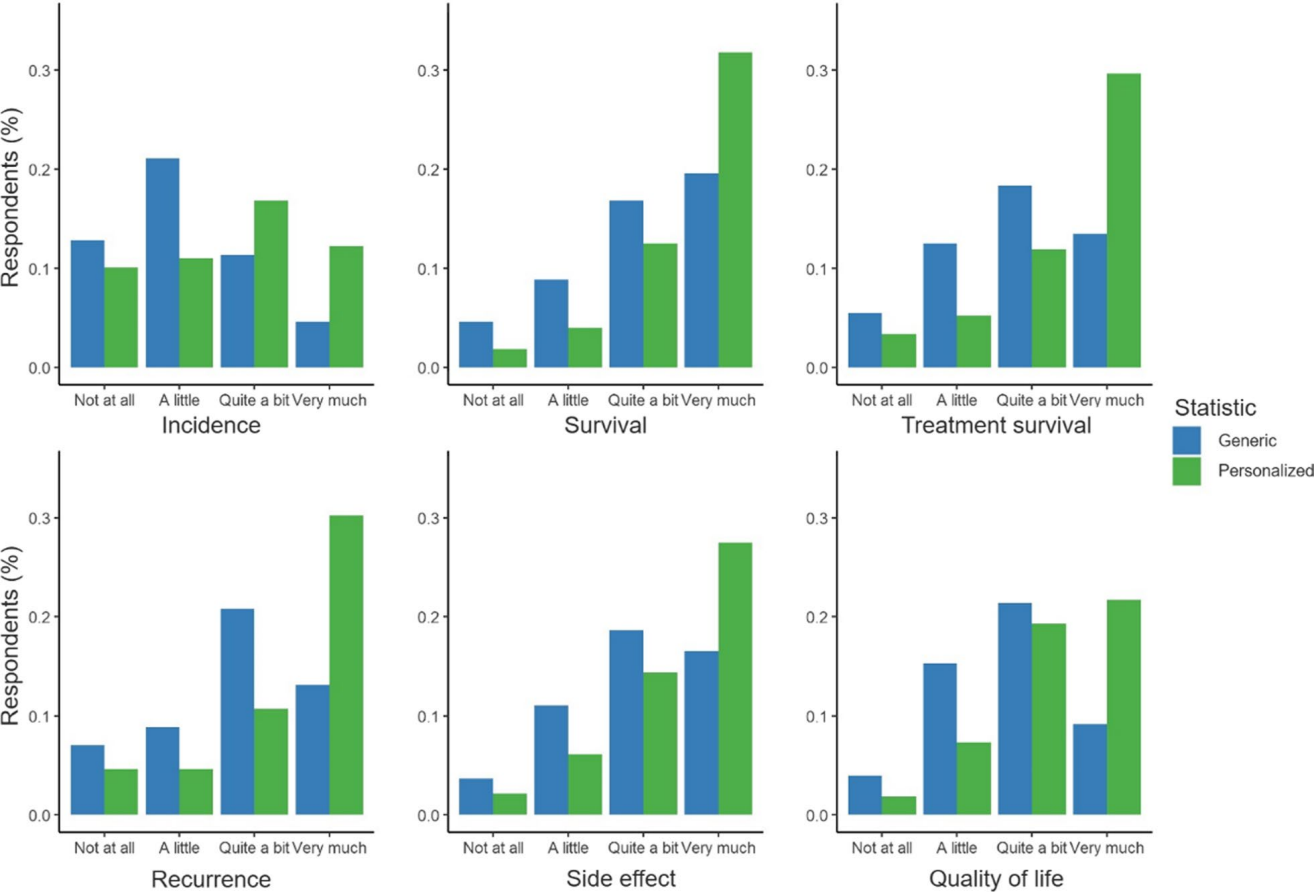
Distribution of needs scores for generic and personalized statistics across topics

Overall, there was a need for both personalized statistics (*M* = 3.14, *SD* = 0.73), *M*_*dif*_ = 1.14, *t*(173) = 20.63, *p* < 0.001, *d* = 1.56, 95% CI [1.04,1.25], and generic statistics^3^ (*M* = 2.70, *SD* = 0.72), *M*_*dif*_ = 0.70, *t*(173) = 12.74, *p* < 0.001, *d* = 0.97, 95% CI [0.59,0.81]. For each topic, there was a stronger need for personalized than for generic statistics (all *p-*values < 0.001, Table 2). Cancer survivors expressed the highest need for receiving the personalized non-treatment related survival rate and risk of treatment side effects, and the lowest need for the generic cancer incidence statistic. Based on distribution scores (Fig. 3), there was a clear preference for personalized over generic statistics (with variation in interest for different topics), but there were also some survivors who did not want anything (but even those would rather have personalized than generic numbers).

**Table 2:** Cancer survivors’ needs for personalized and generic statistics (mean and standard deviations), compared for each topic

Topic	Type of statistic ^a^		*t*	*df*	*d*	95% CI
	Personalized	Generic				
Cancer incidence	2.60 (1.05)	2.15 (0.90)	7.25*	173	0.55	[0.34, 0.60]
Survival rate (non-treatment related)	3.38 (0.84)	2.94 (0.94)	7.20*	172	0.56	[0.35, 0.60]
Survival rate (treatment-related)	3.27 (0.95)	2.75 (0.96)	7.96*	169	0.61	[0.42, 0.68]
Recurrence rate	3.26 (0.98)	2.75 (0.98)	8.20*	166	0.65	[0.40, 0.64]
Risk of side effects	3.32 (0.87)	2.94 (0.93)	6.51*	165	0.51	[0.28, 0.50]
Quality of life	3.13 (0.81)	2.69 (0.81)	8.56*	162	0.66	[0.35, 0.54]

^a^ = Items were rated on a 4-point scale (1 = ‘not at all’, 2 = ‘a little’, 3 = ‘quite a bit’, 4 = ‘very much’); *p < .001

Most cancer survivors (56%) preferred to receive personalized statistical information from their physician, as well as from the internet (*n* = 97), whereas 25% (*n* = 44) preferred to receive this from their physician only, and 16% (*n* = 28) via the internet only. Furthermore, there were no difference in statistical information needs according to time since initial diagnosis, for both generic (*t*(172) = − 0.027, *p* = 0.979, *M*_dif_ = − 0.003, 95% CI [− 0.22,0.11]) and personalized statistics (*t*(172) = − 0.181, *p* = 0.409, *M*_dif_ = − 0.020, 95% CI [− 0.24,0.20]).

### Associations with information coping style, subjective numeracy, and anxiety level

Cancer survivors’ needs for personalized statistics was positively associated with their need for generic statistics (*r* = 0.67, *p* < 0.001). With regard to the information coping style (*M* = 3.01, *SD* = 0.53), survivors who scored higher (information-seekers) had a higher need for personalized (*r* = 0.41, *p* < 0.001) and generic (*r* = 0.37, *p* < 0.001) statistics than participants who scored lower (information-avoiders). Furthermore, the need for personalized statistics was positively related with subjective numeracy (*M* = 4.73, *SD* = 0.97; *r* = 0.29, *p* < 0.001). There was no significant association between the need for generic statistics and subjective numeracy (*r* = 0.11, *p* = 0.181). Additionally, there was no significant association between survivors’ anxiety level (*M* = 5.33, *SD* = 4.02) and their need for personalized statistics (*r* = − 0.05, *p* = 0.564) nor with their need for generic statistics (*r* = − 0.07, *p* = 0.409).

### Statistical need profiles

**Fig. 4:**
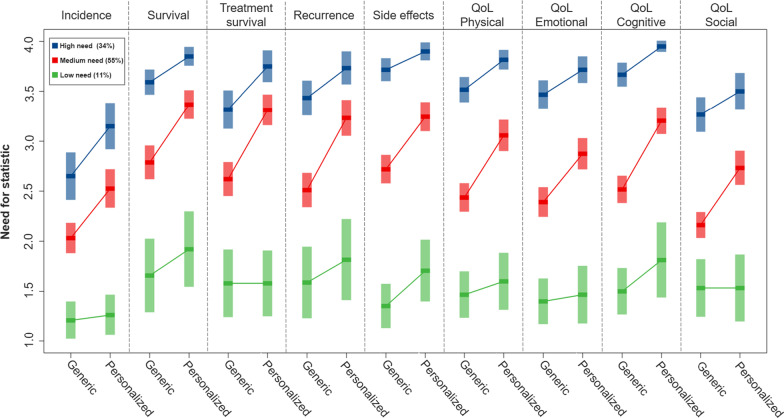
Statistical need profiles for the three classes identified using latent class analysis. The x-axis indicates the need for generic and personalized statistical information, separated for each statistical topic (QoL = Quality of Life). The y-axis indicates respondents’ needs score, measured on a 4-point scale (1 = ‘not at all’, 2 = ‘a little’, 3 = ‘quite a bit’, 4 = ‘very much’). For each class, means and 95% confidence intervals are shown

With the exploratory LCA, three SIN profiles were identified (Fig. 4). Survivors in the first SIN profile (“high SIN”) had a strong need for both generic and personalized statistics (*n* = 60; 34.0%), for each SIN topic (except for incidence rate). The biggest group of survivors are in the second profile (“medium SIN”, *n* = 95, 55.0%), in which survivors had “a little/quite a bit of” need for generic statistics and “quite a bit” of need for personalized statistics. Survivors in the third profile (“low SIN”, *n* = 19, 11.0%) showed “a little” need for both generic and personalized statistics.

Across all profiles, personalized statistics were valued as more important than generic statistics. Additionally, information provided on incidence and social functioning scored lowest on both generic and personalized SIN. There were significant differences in information coping style between the classes, with the highest scores in the first profile (indicating information-seekers), followed by the second profile, and the third profile (Wald = 24.03, *p* < 0.001). We observed no significant differences in terms of sociodemographic characteristics, clinical characteristics, anxiety level, and numeracy skills (see Additional File 1 for characteristics of and comparisons between SIN profiles).

### Exploring views on statistical information needs

Based on comments from 98 respondents, we identified seven themes that summarize considerations people have for (not) wanting personalized and/or generic statistics. Almost half (*n* = 48) mentioned that receiving personalized statistics would give them a feeling of being somewhat in control in turbulent times. They mentioned it would help them to create a better picture of what life would be like after diagnosis, make plans for the future, better understand their disease, and manage expectations. One participant said:

*“It gives you a tool from which you can be motivated to take action or not. A tool to deal with a situation that is life-threatening.” [Woman aged 53, lung cancer]*

That feeling of wanting to be in control is shared by many of the participants and seems to be related to wanting to be in charge of the decision-making process. Many note the importance of receiving (specific) numbers to make informed decisions about treatments, but also decisions after treatments can be based on this kind of information:

*“[…] You want to sort of remain in control of your life and be prepared. If I know that I have an 80 percent chance of being alive 15 years after diagnosis, then I feel more at ease than knowing it’s only 30 percent. This also causes you to make different decisions.” [Woman aged 48, breast cancer]*

Some also commented on the difference between personalized and generic statistics (*n* = 17). Many wanted to receive both types of statistics in order to compare them. This would help them with interpreting the numbers better and feeling even more in control about their own life after diagnosis.

*“I need the generic statistics to put my personalized statistics into perspective.” [Man aged 65, lung cancer]*

Although many might want personalized statistics, some also comment on the (un)availability of data and the tough spot they are in because of that (*n* = 9). As one participant put it:

*“In 1995, these data were unavailable. There were only data about strictly medical consequences of amputation and radiation ... There is much more information now and I think that could have helped me to - with the social, emotional and societal issues I ran into because of the cancer – not ask myself again and again where all of these issues came from.” [Woman aged 58, breast cancer]*

The importance of receiving more personalized statistics is also stressed by this participant:

*“Because I am relatively young to have rectum cancer, I have the idea that the numbers are not totally representative for my situation. Because, how much percent of people die from underlying issues? If you are 70 and you add 5 years, then the chances of dying are higher anyway than for someone who is 40 … That’s why I would find it very useful to know the numbers aimed at my age group.” [Woman aged 38, colon cancer]*

There were some people who were dissatisfied with the statistics they were given (*n* = 6). For example, one participant noted:

*“I would really like to know what my chances are. Doctors give me little specific information, but only generic information. I did ask for it though, but I never received any answers. It almost looks like they can’t say anything about it. That’s very frustrating.” [Man aged 71, prostate cancer]*

There was a small group of people that can be classified as statistics-lovers (*n* = 13), who commented that they prefer numbers rather than words by saying:

*“The words ‘little’ or ‘rarely’ do not tell me anything. Percentages tell me a lot more and are more specific.” [Woman aged 59, lung cancer]*

*“The more information I receive, the better. Information in terms of numbers is typically short and powerful and tells me more than just words.” [Man aged 74, prostate cancer]*

In contrast, there was also a group of people that did not want specific numbers at all (*n* = 15), for instance because they felt the numbers did not tell them much since “everybody is unique”. Or, as one participant put it:

*“I’m not really fond of predictions or results, every person is different and what happens to you happens to you … nothing you can do about it.” [Woman aged 68, colon cancer]*

Additionally, some participants had negative experiences with statistics, or they did not want to know everything about their future because they “live day by day”. This seems especially true for those who had metastatic cancer:

*“In my process, statistics often gave a wrong indication, both in a positive and in a negative way. With that, the available numbers have created a false (un)certainty, which is there still.” [Man aged 54, colon cancer]*

*“Personally, I would not want specific numbers. I have metastatic prostate cancer. The PSA-levels are increasing, but I remain positive and optimistic. I would absolutely not want to know what my expectations are or the remaining time I still possibly have. Now I can live with this quite well and would absolutely not want that this whole situation would affect my emotions.” [Man aged 63, prostate cancer]*

## Discussion

Our findings highlight that most people in our selective sample diagnosed with cancer want to receive statistical information on different health outcomes [19, 20, 31], and especially personalized statistics adjusted to their personal and tumor characteristics [22, 48]. However, currently such statistics are not always personalized in clinical practice and patient decision aids [16–18]. In line with previous research [20, 31], personalized survival outcomes, risks of side effects, and recurrence rates are deemed most relevant by patients or cancer survivors, followed by quality of life statistics. Ironically, survivors showed little need for the cancer incidence statistic, while this number is communicated the most in patient decision aids [16–18]. As such, there seems to be a discrepancy in what patients actually want to receive and what they often get.

Furthermore, information-seekers expressed a stronger need for both personalized and generic statistics than information-avoiders. This highlights the importance of exploring patients’ information coping style when deciding to disclose (personalized) numerical data with patients [21]. Indeed, patients typically report better quality of life and less anxiety if their information needs are congruent with what they received [49]. The association between SIN and subjective numeracy was partly found; survivors with higher subjective numeracy showed more need for receiving personalized statistics, but not for generic statistics. This indicates the importance of distinguishing between these two types of statistics. It seems that people who perceive themselves as being good with numbers also view personalized numbers as more important, while those lower in subjective numeracy may not seek out individualized numeric data, possible due to its difficulty and/or emotional reactions to them [28]. Future studies could focus on whether more subjectively numerate patients also estimate their risks more accurately when receiving personalized statistics.

No association was found between anxiety and patient need for statistical information. Some studies found that patients who are more anxious may have lower needs in receiving statistical information that is too anxiety provoking (e.g., unfavorable survival or recurrence rates) which can help them preserve hope [19, 20]. However, others found the opposite, by showing that patients with higher anxiety scores wanted to know more prognostic information [29]. Since we measured how anxious people felt in the past two weeks, it could still be that receiving personalized numbers affects anxiety induced by the personalized format. One might argue that the group most at risk for induced anxiety levels are those that receive the worst news. However, researchers have demonstrated that most metastatic cancer patients prefer to have as much information as possible, regardless of the severity of the outcome [13, 50–52]. More effect studies could help identify the boundaries of providing personalized statistics, especially when their personalized outcome paints a worse picture than the generic outcome [53].

In addition, we identified three statistical need profiles based on cancer survivors’ answers on the SIN items. Besides the well-known distinction between the information-seeker (“high SIN”; 34%), characterized by a strong need for both personalized and generic statistics, and the information-avoider (“low SIN”; 11%), characterized by low statistical information needs, a third group showed to be the largest group within our sample of cancer survivors. This group (“medium SIN”; 55%) showed a somewhat different pattern, characterized by a medium need for generic statistics, but a strong need for personalized statistics. Survivors with both a strong need for personalized and generic statistics were characterized by a high information-seeking coping style. Our findings build on existing studies that identified patient profiles based on information needs [30, 54], and also show that the majority of our sample want to receive statistics related to personalized treatment outcomes.

Our study also explored reasons patients might have for preferring (personalized) statistics. Almost half of our sample commented that personalized statistics would let them feel more in control. This could be explained by the ‘locus of control’ theory [55], which refers to “the perception that events are determined by one's own behavior (internal control) or by such outside forces as other people or fate (external control)” [56]. Even though patients were diagnosed with cancer (external control), receiving personalized statistics could lead to patients feeling more empowered and actively involved in the decision-making process (internal control). Research has shown that experiencing internal control can have a positive impact on how anxious or depressed people feel [57]. With respect to people who want to receive both generic and personalized statistics to compare information, research has highlighted the positive effects of including such comparative risk information [58, 59], although the effects of including comparative risk information may vary between contexts and individuals [53, 60]. Finally, to shed more light on people who have a low need for receiving statistics, some patients with metastatic expressed no need for statistics, as they would feel less motivated. However, this is not automatically true for all metastatic cancer patients as many still want to be thoroughly informed [29]. Taken together, this explorative analysis calls for a more in-depth interview study on the reasons why patients might not want to receive personalized (statistical) information.

### Strengths and limitations

This study provides the first comprehensive assessment of cancer survivors’ needs for receiving statistics after diagnosis, while distinguishing between generic and personalized statistics. However, an important limitation relates to our sample, which was relatively small and consisted of (active) cancer survivors involved in online cancer communities or patient organizations. This selection may not represent the general cancer population, as they are educated and demonstrate higher levels of internet use [61, 62], which may impact the generalizability of our results. However, it is interesting to note that there were still clear differences within this selective group of cancer survivors. For instance, with regards to information coping style there was still a group of blunters (i.e., information-avoiders) in our sample. Moreover, patients who have survived cancer may have different perspectives on receiving risk information compared to those who are (newly) diagnosed with cancer and/or are undergoing treatment, for instance for receiving prognostic information [21]. For ethical reasons, we included cancer survivors in our sample since we did not want to interfere with the current information provision for (newly) diagnosed patients, especially with those who may not yet receive personalized numerical information. Nevertheless, increasing evidence suggests that providing newly diagnosed patients with personalized numerical information “is not to be feared” and may positively contribute to shared decision-making [63–65]. In order to gain a comprehensive assessment of the statistical needs of cancer patients, future research should be inclusive of the full range of (newly diagnosed) cancer patients.

Furthermore, we did not focus on *how* patients want to receive such information (e.g., verbal, numerical, visual) [66]. Especially since cancer survivors wanted to receive personalized statistics about quality of life in a numerical format, more research should be dedicated to how to present such subjective data [67]. We also bear in mind that we measured subjective numeracy rather than objective numeracy. Although the two concepts are highly related [40], subjective numeracy also takes into account how people feel about their skills so there is a possibility people over- or underestimate their numerical abilities.

Additionally, in our study we assumed that data would be readily available for all of the topics and cancer types, while this is not necessarily the case in clinical practice. Moreover, understanding uncertainty around statistics is challenging, especially when communicating personalized statistics as reference groups decrease [6]. This, in turn, means that a personalized risk might be less reliable from a statistical perspective. However, even simple patient characteristics (‘tumor type’ or ‘age’) could be used to personalize outcomes [67] and most studies on communicating personalized risks for cancer screening found positive results [68]. What the effects are of discussing personalized risks about side effects, diagnosis or quality of life in general should be studied more thoroughly, but individual patient tools that communicate personalized risks about cancer could yield positive results [9, 69, 70]. Finally, in line with current practices, the cancer incidence statistic was the only statistic that was not presented as a rate, which could be a reason for the lower interest.

### Implications

Our results are encouraging for research into patient needs with respect to personalized information provision and the disclosure of health risk data [67, 71, 72]. Most cancer survivors in our sample reported a strong need for receiving personalized statistics on different topics, ranging from survival rates to quality of life information. In practice, the need for personalized statistics can change depending on phase of the disease, with newly diagnosed patients wanting (personalized) statistics on survival, patients in the decision-making stage wanting such numbers for side effects and risk of recurrence and patients after the treatment phase wanting information on quality of life [73]. Our results are also useful for further development and implementation of data-driven personalized decision aids and (web-based) risk prediction models in oncology [67, 71, 72, 74, 75]. Moreover, the empirical findings contribute to the rapidly expanding fields of personalized medicine [76], individualized medical decision-making [5], patient-centered care, and shared decision-making [2]. As some participants reported, personalized statistics should not replace generic statistics, but instead could be communicated in combination according to patient needs and preferences. This way, patients can make better sense of the personalized statistics and learn how they compare to the average, population-based statistics [77].

The findings also shed light on possible contributing factors such as a patient’s information coping style or subjective numeracy. Based on our qualitative analysis, we can see that patients might want personalized statistics, both personalized and generic statistics, or no statistics at all. By asking individual patients if they would want to receive (personalized) statistics, nurses and clinicians could empower patients to become more aware of the kind of role they want to play in their decisions. Our results suggest that a patient’s information coping style could be an important indicator if both generic and personalized statistics should be provided. Additionally, people with high subjective numeracy also express a stronger need for personalized statistics. Both characteristics of patients could be part of an online decision aid that patients fill out before entering a consultation, so that healthcare professionals can effectively tailor the type of statistics that they want (or do not want) to disclose to individual patients.

## Conclusions

We found that the majority of our sample of cancer survivors expressed a strong need for receiving personalized statistics on different topics during treatment decision-making. Information coping style and subjective numeracy seem to be important factors for determining whether a patient wants to receive personalized statistical information. Our results encourage further development and implementation of data-driven personalized decision aids and risk prediction models in oncology practice care to help patients making well-informed and shared decisions about treatment.

## Supplementary Information

**Additional file 1**. Characteristics of and comparisons between statistical information needs (SIN) profiles.

## Data Availability

The dataset generated and/or analyzed during the current study are available in the Open Science Framework repository, https://osf.io/qv35z/.

## Footnotes

1 Based on patients’ feedback on our questionnaire during the pre-test, we made the following changes. We first added two questions: (1) “how satisfied are you with the information you received during your treatment process” as some of the patients mentioned they were already satisfied and this may affect their need for personalized statistics, and (2) “Imagine that you would be able to receive these specific numbers, how would you want to receive them? 1: Through my doctor during a consultation, 2: Through the internet, 3: Both through my doctor during a consultation as well as via the internet, 4: I do not want to receive specific numbers.”, as this might influence their willingness to receive personalized statistics. Second, we added an explanation to the control question: “Mind you, for the sake of this research we presume that all data are available. Even if you have a rare form of cancer, we are interested to know if you have a need for these specific numbers.”, as one of the pre-tested patients noted that they had a rare form of cancer so questions might not apply to them. Finally, we revised the phrasing of some questions (e.g., for the demographic question on work status “incapacitated” was changed to “temporarily incapacitated”, and for the 5-year-recurrance items that accidentally talked about “1-year recurrence” was changed into “5-year recurrence”).

2 When we started with the design of our SIN-questionnaire, we initially used a 5-point scale (ranging from 1 as “none at all” to 5 as “very much”). However, after careful expert evaluation we thought it would be better to use a 4-point scale as it would be clearer what the score “2” meant. We also included verbal meanings to the scales (i.e., “1: not at all”, “2: a little”, “3: quite a bit” to “4: very much”) for each item, to help respondents better interpret the answer options. Unfortunately, we mistakenly still put a test-value of 3 in our pre-registration within the Open Science Framework. As our aim was to identify whether or not people have a need for personalized and generic statistics, a test score of 2 would be better since “a little” need already indicates that there is a need. We therefore changed our pre-registered analysis.

3 There was a medium ordering effect of the need for general statistics (*t*(159) = 2.02, *M*_dif_ = 0.23, *p* = .045, *d* = .31, 95% CI [0.01,0,45]) with people who answered questions about generic statistics before personalized statistics scoring higher on their general statistical needs (*M* = 2.83, *SD* = 0.68) than people who answered questions about personalized statistics first (*M* = 2.61, *SD* = 0.75). Since distribution between conditions was equal, this did not impact the results.
