# Supplementary material for: Need for numbers: assessing cancer survivors’ needs for personalized and generic statistical information
Source: BMC Med Inform Decis Mak. 2022 Oct 5;22:260. doi: 10.1186/s12911-022-02005-2 (PMC9535944; doi:10.1186/s12911-022-02005-2)
Supplement: Supplementary file 1 — Additional file 1. Characteristics of and comparisons between statistical information needs (SIN) profiles. [file 12911_2022_2005_MOESM1_ESM.docx]

**Additional file 1**

**Title:** Characteristics of and comparisons between statistical information needs (SIN) profiles.

**Description:** Characteristics of SIN profiles (Table A1) in terms of demographic variables and individual difference measures (including information coping style, subjective numeracy, and anxiety level). Furthermore, Table A2 displays comparisons between the SIN profiles in terms of demographic variables and individual difference measures (including information coping style, subjective numeracy, and anxiety level).

**Table A1.** Characteristics of SIN^a^ profiles in terms of demographic variables and information coping style, numeracy skills and anxiety level.

|  | **Profile 1**  **“High SIN”**  ***n* = 60** | **Profile 2  “Medium SIN”**  ***n* = 95** | | **Profile 3 “Low SIN”**  ***n* = 19** | | |
| --- | --- | --- | --- | --- | --- | --- |
| **Gender** |  |  |  |  |  |  |
| Male | 26 (43.3%) | 40 (42.1%) | | 5 (26.3%) | | |
| Female | 34 (56.7%) | 55 (57.9%) | | 14 (73.7%) | | |
| **Age** |  |  |  |  |  |  |
| Mean (SD) | 60.2 (7.7) | 60.3 (9.9) | | 59.3 (9.9) | | |
| **Education** |  |  |  |  |  |  |
| Low | 5 (8.3%) | 7 (7.4%) | | 3 (15.8%) | | |
| Medium | 13 (21.7%) | 37 (38.9%) | | 9 (47.4%) | | |
| High | 42 (70.0%) | 51 (53.7%) | | 7 (36.8%) | | |
| **Marital status** |  |  |  |  |  |  |
| Married /Partner | 51 (85.0%) | 72 (75.8%) | | 17 (89.5%) | | |
| No partner | 9 (15.0%) | 23 (24.2%) | | 2 (10.5%) | | |
| **Tumor** |  |  |  |  |  |  |
| Breast | 26 (43.3%) | 34 (35.8%) | | 7 (36.8%) | | |
| Colorectal | 8 (13.3%) | 26 (27.4%) | | 6 (31.6%) | | |
| Lung | 7 (11.7%) | 11 (11.6%) | | 3 (15.8%) | | |
| Prostate | 19 (31.7%) | 24 (25.2%) | | 3 (15.8%) | | |
| **Years since diagnosis** |  |  |  |  |  |  |
| Mean (SD) | 6.1 (6.2) | 5.9 (11.6) | | 5.3 (5.5) | | |
| **Information coping style** |  |  |  |  |  |  |
| Mean (SD) | 5.8 (6.3) | 1.9 (6.1) | | -2.5 (6.7) | | |
| **Anxiety level** |  |  |  |  |  |  |
| Mean (SD) | 5.1 (4.5) | 5.5 (3.7) | | 5.3 (4.1) | | |
| **Subjective numeracy** |  |  |  |  |  |  |
| Mean (SD) | 4.9 (1.0) | 4.7 (0.9) | | 4.2 (1.0) | | |

^a^ SIN = Statistical Information Need.

**Table A2.** Comparisons between the SIN^a^ profiles in terms of demographic variables and information coping style, numeracy skills and anxiety level.

| **Profiles (Ref = Profile 2 “Medium SIN”)** | **Profile 1 “High SIN” (95% CI)** | **Profile 3 “Low SIN” (95% CI)** | | | **Wald** | | ***p*-value** | |
| --- | --- | --- | --- | --- | --- | --- | --- | --- |
| **Gender (Ref = Male)** |  |  |  |  |  | | |  |
| Female | -0.08 (-0.76; 0.59) | 0.75 (-0.38; 1.88) | | | 2.028 | | .360 | |
| Age | -0.08 (-2.93; 2.77) | -1.13 (-5.95; 3.70) | | | 0.219 | | .900 | |
| **Education (Ref = Low)** |  |  |  |  |  |  | | |
| Medium | -0.76 (-2.11; 0.60) | -0.59 (-2.18; 1.00) | | | 7.926 | | .094 | |
| High | 0.01 (-1.26; 1.28) | -1.25 (-2.88; 0.38) | | |  | |  | |
| **Marital status (Ref = Married /Partner)** |  |  |  |  |  |  | | |
| No partner | -0.55 (-1.43; 0.32) | -1.01 (-2.61; 0.59) | | | 2.564 | | .280 | |
| **Tumor (Ref = Breast)** |  |  |  |  |  |  | | |
| Colorectal | -0.90 (-1.89; 0.09) | 0.10 (-1.12; 1.32) | | | 6.324 | | .390 | |
| Lung | -0.36 (-1.50; 0.79) | 0.21 (-1.32; 1.75) | | |  | |  | |
| Prostate | 0.16 (-0.66; 0.97) | -0.50( -1.98; 0.98) | | |  | |  | |
| **Year since diagnosis** | 0.20 (-2.81; 3.20) | -0.51 (-3.99; 2.97) | | | 0.227 | | .890 | |
| **Information coping style** | 3.93 (1.79; 6.07)* | -4.64 (-8.49; -0.80)* | | | 24.032 | | <.001 | |
| **Anxiety level** | -0.33 (-1.78; 1.13) | -0.15 (-2.49; 2.20) | | | 0.193 | | .910 | |
| **Subjective numeracy** | 0.16 (-0.18; 0.50) | -0.50 (-1.06; 0.07) | | | 4.901 | | .086 | |

^a^ SIN = Statistical Information Need.
